# Levels of plasma circulating cell free nuclear and mitochondrial DNA as potential biomarkers for breast tumors

**DOI:** 10.1186/1476-4598-8-105

**Published:** 2009-11-17

**Authors:** Corina Kohler, Ramin Radpour, Zeinab Barekati, Reza Asadollahi, Johannes Bitzer, Edward Wight, Nicole Bürki, Claude Diesch, Wolfgang Holzgreve, Xiao Yan Zhong

**Affiliations:** 1 Laboratory for Prenatal Medicine and Gynecologic Oncology, Women's Hospital/Department of Biomedicine, University of Basel, Switzerland; 2 Kantonsspital Liestal, Frauenklinik, Switzerland; 3 University Medical Centre of Freiburg, Germany

## Abstract

**Background:**

With the aim to simplify cancer management, cancer research lately dedicated itself more and more to discover and develop non-invasive biomarkers. In this connection, circulating cell-free DNA (ccf DNA) seems to be a promising candidate. Altered levels of ccf nuclear DNA (nDNA) and mitochondrial DNA (mtDNA) have been found in several cancer types and might have a diagnostic value.

**Methods:**

Using multiplex real-time PCR we investigated the levels of ccf nDNA and mtDNA in plasma samples from patients with malignant and benign breast tumors, and from healthy controls. To evaluate the applicability of plasma ccf nDNA and mtDNA as a biomarker for distinguishing between the three study-groups we performed ROC (Receiver Operating Characteristic) curve analysis. We also compared the levels of both species in the cancer group with clinicopathological parameters.

**Results:**

While the levels of ccf nDNA in the cancer group were significantly higher in comparison with the benign tumor group (*P *< 0.001) and the healthy control group (*P *< 0.001), the level of ccf mtDNA was found to be significantly lower in the two tumor-groups (benign: *P *< 0.001; malignant: *P *= 0.022). The level of ccf nDNA was also associated with tumor-size (<2 cm vs. >2 cm<5 cm; 2250 vs. 6658; Mann-Whitney-U-Test: *P *= 0.034). Using ROC curve analysis, we were able to distinguish between the breast cancer cases and the healthy controls using ccf nDNA as marker (cut-off: 1866 GE/ml; sensitivity: 81%; specificity: 69%; *P *< 0.001) and between the tumor group and the healthy controls using ccf mtDNA as marker (cut-off: 463282 GE/ml; sensitivity: 53%; specificity: 87%; *P *< 0.001).

**Conclusion:**

Our data suggests that nuclear and mitochondrial ccf DNA have potential as biomarkers in breast tumor management. However, ccf nDNA shows greater promise regarding sensitivity and specificity.

## Introduction

In several branches of biomedical research the quest for new disease-related biomarkers has become one of the main objectives [1-3]. When it comes to discover and develop new biomarkers, oncology seems to be the most ambitious field. During the last few years a lot of research has been done identifying new cancer biomarkers with the aim to identify high risk individuals, detect cancer at an early stage, predict outcome, monitor treatment and screen for disease recurrence [4]. In this respect the focus is now mainly directed towards the identification of non-invasive cancer biomarkers [5,6].

In the case of breast cancer, there are only a few non-invasive biomarkers for screening, predicting prognosis and monitoring that have come to routine clinical application [7]. Current established methods for routine breast cancer screening firstly encompass non-invasive methods including clinical breast examination and imaging techniques like mammography and ultrasonography [8]. However, when pathological changes are suspected these techniques generally have to be followed by histopathological analysis for which invasive procedures, such as biopsies, are needed.

Lately, the discovery of circulating cell-free DNA (ccf DNA) has sparked the interest of scientists as it opens up a new possibility for non-invasive analysis of tumor derived genetic material. Both ccf nuclear DNA (nDNA) and mitochondrial DNA (mtDNA) have become a matter of investigation and qualitative as well as quantitative alterations in these two determinants have been implicated in cancer [9]. Changes in the level of ccf nDNA and mtDNA have been found in plasma and serum of patients with various cancer types [10,11]. In breast cancer patients it has been shown that ccf nDNA levels are elevated in plasma as well as in serum when compared to healthy controls [12,13]. On the other hand, mtDNA levels were mostly found to be decreased in breast cancer patients in comparison to healthy controls [14,15].

To investigate the potential of ccf nuclear and mitochondrial DNA as a marker for clinical application we examined the level of both species in malignant and benign tumor groups and healthy controls.

## Materials and methods

The study was performed at the Laboratory for Prenatal Medicine and Gynecological Oncology/Department of Biomedicine, Women's Hospital Basel and approved by the local institutional review board (Ethic commission beider Basel). Written consent forms were collected from all patients who were involved in this study.

### Study cohort and sampling procedure

The blood samples used in this study were collected in a time period from 2005 to 2007 in either the Women's Hospital of the University of Basel or the Women's Hospital of Liestal. In total 148 women were included in the study. Most of the women were European Caucasians. All blood samples were taken before any surgical interventions or therapeutic treatments. Patients' data (age, tumor size, lymph node involvement, extent of metastasis, estrogen receptor, progesterone receptor and Her2neu - status) were obtained from the pathological reports. The blood samples were processed and the DNA was extracted according to a standardized protocol as previously described elsewhere [16]. DNA was quantified using a Nanodrop spectrophotometer (Thermo scientific).

The study cohort (n = 148) was divided into 3 groups: 1) malignant disease group (n = 52); 2) benign disease group (n = 26) and 3) healthy control group (n = 70). For groups 1 and 2 the diagnoses were all biopsy-confirmed. The healthy control group used in this study neither had a history of cancer nor suffered from any other severe diseases.

### qPCR

For the simultaneous quantification of ccf nDNA and mtDNA from plasma a multiplex qPCR was performed using the Glyceraldehyd-3-phosphat-dehydrogenase (*GAPDH*) and the mtDNA encoded ATPase 8 (*MTATP 8*) reference genes.

The gene IDs, the amplicon length, the annealing temperature and the sequence information of primers and probes for the *GAPDH *and the *MTATP 8 *reference genes are shown in table 1.

**Table 1:** Quantitative PCR (qPCR) for *GAPDH *and *MTATP8*

Gene	Gene ID	Sequences of primers and probes (5' → 3')		Length of primer/probe	Amplicon lengths (bp)
*GAPDH*	2597	Forward	CCC CAC ACA CAT GCA CTT ACC	21	97
			
		Reverse	CCT AGT CCC AGG GCT TTG ATT	21	
			
		Probe	(MGB) TAG GAA GGA CAG GCA AC (VIC)	17	

*MTATP8*	4509	Forward	AAT ATT AAA CAC AAA CTA CCA CCT ACC	27	78
			
		Reverse	TGG TTC TCA GGG TTT GTT ATA	21	
			
		Probe	(MGB) CCT CAC CAA AGC CCA TA (FAM)	17	

Quantitative PCR (qPCR) for *GAPDH *and *MTATP8 *was carried out in a total reaction volume of 25 μl containing 7 μl H_2_O, 12.5 μl TaqMan^® ^Universal PCR Master Mix, 0.75 μl of each of the shown 10 μM primers, 1 μl of a 5 μM FAM-labeled *MTATP 8*-probe and 0.5 μl of a 5 μM VIC-labeled *GAPDH*-probe and 1 μL of template. The reaction was performed at the following conditions: initiation for 2 minutes at 50°C, followed by a first denaturation for 10 minutes at 95°C and a further step consisting of 40 cycles of 15 seconds at 95°C and 1 minute at 60°C.

qPCR was carried out in 25 μl of total reaction volume containing 7 μl H_2_O, 12.5 μl TaqMan^® ^Universal PCR Master Mix (Applied Biosystems, Branchburg, New Jersey, USA), 0.75 μl of each of the above mentioned 10 μM primers (Microsynth, Balgach, Switzerland), 1 μl of a 5 μM FAM-labeled *MTATP 8*-probe and 0.5 μl of a 5 μM VIC-labeled *GAPDH*-probe (both probes from Applied Biosystems, Rotkreuz, Switzerland). For each reaction 1 μl of DNA was added. qPCR was performed using the ABI PRISM 7000 Sequence Detection System (Applied Biosystems, Branchburg, New Jersey, USA) under the following conditions: an initiation step for 2 minutes at 50°C is followed by a first denaturation for 10 minutes at 95°C and a further step consisting of 40 cycles of 15 seconds at 95°C and 1 minute at 60°C.

### Data collection and processing

The threshold cycle (Ct) values were obtained by the ABI Prism 7000 software. Each sample was analyzed in duplicate and one negative control was included in every run. For calibration a standard calibrator curve with known genomic DNA concentrations ranging from 3.125 × 104 to 10 pg/μL with a dilution factor of 5 (including 31250, 6250, 1250, 250, 50 and 10 pg/μL) was used. The efficiency of the multiplex assay for amplifying both nDNA and mtDNA simultaneously was measured in our previous study using standard curves generated by dilution series [17]. The standard curves had average slopes at approximately -3.3 (~100% efficiency). *GAPDH *and *MTATP8 *levels were normalized by data obtained from the amplification of HPLC-purified single-stranded synthetic DNA oligonucleotides (Microsynth) specifying a 97-bp *GAPDH *amplicon and a 79-bp *MTATP8 *gene amplicon with concentrations ranging from 5 × 10^7 ^copies to 5 × 10^2 ^copies. The concentrations of ccf nDNA were calculated according to the standard curves, using known concentration of human genomic DNA. The results were expressed as genome-equivalent (GE) per mL of plasma by using the conversion factor of 6.6 pg of DNA per cell. Genome equivalents were calculated as follows:

For the calculation of the concentration (c) in genome equivalents (GE/mL) the DNA quantity (Q) obtained by qPCR was multiplied with one fraction consisting of the volume of eluted DNA (V_DNA; _80 μl/sample) divided by the sample volume used for PCR (V_PCR_; 2.5 μl/reaction) resulting in a factor of 32 and with another fraction consisting of the unit (1 ml) divided by the volume of extracted plasma (V_EX _= 400 μl) resulting in a factor of 2.5.

The content of mtDNA was calculated using the delta Ct (ΔCt) of an average Ct of mtDNA and nDNA (ΔCt = CtnDNA - CtmtDNA) in the same well as an exponent of 2 (2Δ^Ct^). Relative quantities of ccf mtDNA could be estimated using an equation of GE (nDNA) × fold-change mtDNA and expressed also as GE per mL of plasma.

### Statistical Analysis

All statistical analyses were performed using SPSS 17.0 (SPSS Inc., Chicago, USA). The normality distribution of the data was determined using the Shapiro-Wilk-Test. The data were not normally distributed. For comparison of ccf nDNA and mtDNA levels between the three groups (malignant disease group, benign disease group and healthy control group) the Mann-Whitney-U-Test was performed. For the comparison of the ccf nDNA and mtDNA levels with other established prognostic factors the Mann-Whitney-U-Test and the Kruskal-Wallis-Test were used. *P*-values ≤ 0, 05 were considered statistically significant.

## Results

### Comparison of plasma ccf nDNA and mtDNA levels between the three study-groups

We compared the levels of plasma ccf nDNA and mtDNA, analyzed by multiplex real-time PCR, between the malignant disease group, the benign disease group and the healthy control group. The level of ccf nDNA in the malignant disease group was significantly higher in comparison with the benign disease group (4678 vs. 1359, Mann-Whitney: *P *< 0.001) and the healthy control group (4678 vs.1298, Mann-Whitney: *P *< 0.001). No significant difference could be found in the level of nDNA between the benign disease group and the healthy controls (1359 vs. 1298 Mann-Whitney: *P *= 0.830).

**Figure 1:**
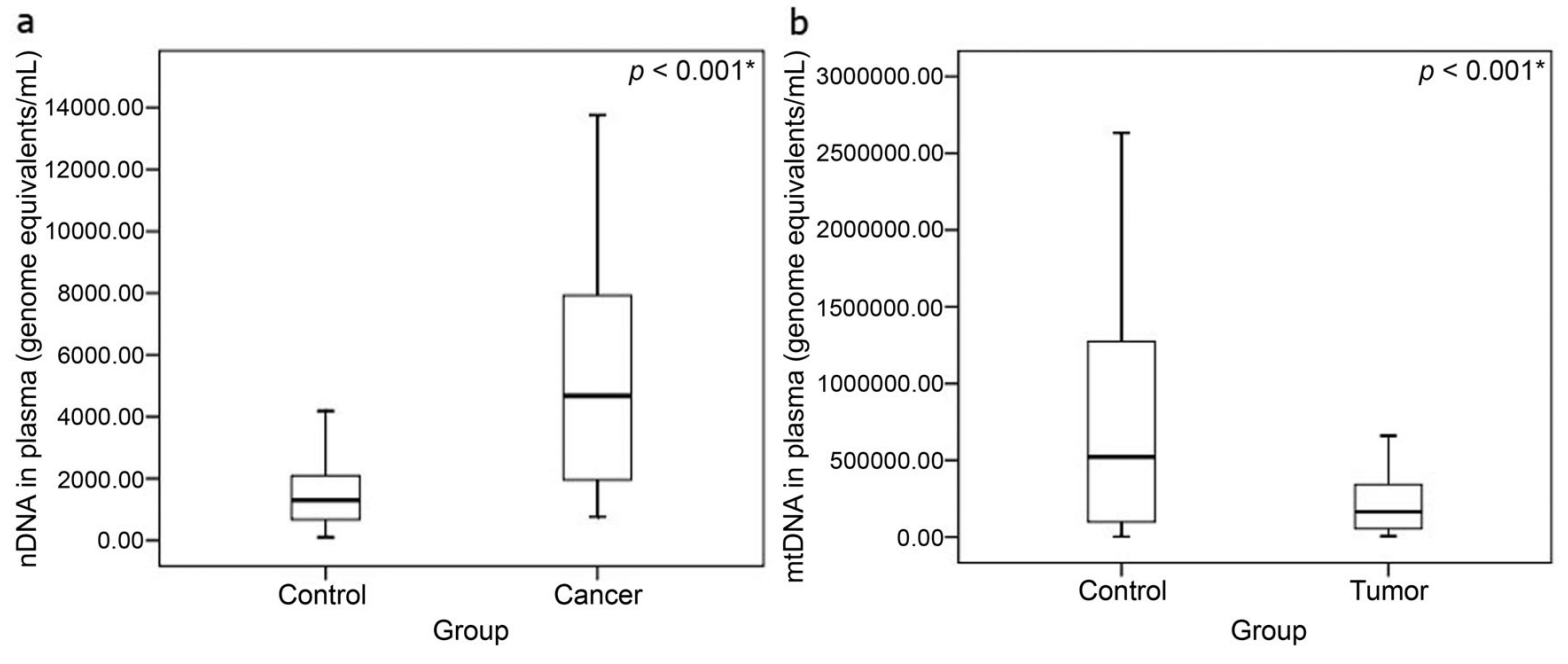
**Boxplot for the comparison of the ccf nDNA and mtDNA levels between the study- groups**. A) Boxplot for comparison of ccf nDNA levels between the malignant disease group and the healthy control group. Level of ccf nDNA in the cancer group was significantly higher in comparison with the healthy control group (*P *< 0.001). As no significant difference was found in the level of ccf nDNA between the benign disease group and the healthy controls the comparison is not shown in the figure. B) Boxplot for comparison of ccf mtDNA levels between the tumor group (including the malignant and benign cases) and the healthy control group (*P *< 0.001). Decreased levels of ccf mtDNA was found in both, the benign disease group and the malignant disease group, when compared to the healthy control group. (* significant correlation; Mann-Whitney-U-Test).

In contrast to the ccf nDNA determination, a decreased level of ccf mtDNA was found in the malignant disease group when compared with the healthy control group (205013 vs. 522115, Mann-Whitney; *P *= 0.022) and the benign disease group (205013 vs. 73977; Mann-Whitney: *P *< 0.001). However, in the benign disease group the level of ccf mtDNA is even significantly lower than in the malignant disease group (73977 vs. 205013; Mann-Whitney: *P *< 0.001). The median of plasma ccf nDNA and mtDNA in the three study groups is shown in Table 2. The comparison of the ccf nDNA and mtDNA levels between the study groups is depicted in Fig. 1.

**Table 2:** Concentrations (GE/mL) of plasma ccf nDNA and ccf mtDNA in the 3 study-groups; expressed as median.

Group	Total no. of patients	Age (mean ± S.D.)	Median Ccf nDNA(GE/mL)	Median Ccf mtDNA (GE/mL)
Malignant disease group	52	64 ± 15	4678	205013
Benign disease group	26	41 ± 16	1359	73977
Control group	70	53 ± 14.6	1298	522115

### Correlation between the level of plasma ccf nDNA and mtDNA with clinicopathological parameters

For the malignant disease group, the association between the level of ccf DNA and other established clinical parameters, including tumor size, lymph node involvement, extent of metastasis and the status of estrogen receptor (ER), progesterone receptor (PR) and Her2/neu were analyzed.

### Association between plasma ccf DNA level and tumor size in the malignant disease group

**Figure 2:**
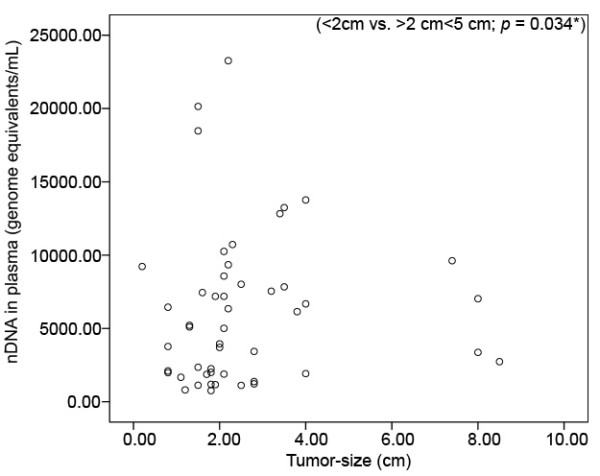
**Scatterplot for correlating levels of ccf nDNA between breast cancer patients with a tumor size > 5 cm; (n = 4), >2 cm<5 cm; (n = 25) and < 2 cm; (n = 21)**. Significant difference in the levels of ccf nDNA could be found between tumors with a tumor size of >2 cm<5 cm and tumors with a tumor size of < 2 cm (*P *= 0.034). For the group of the tumor size > 5 cm, only 4 cases were recruited. (* significant correlation; Mann-Whitney-U-Test).

The level of ccf nDNA was significantly lower in patients with breast cancer with a tumor size of less than two centimeters (<2 cm; n = 21) than in those with a tumor size between 2 and 5 centimeters (>2 cm<5 cm; n = 25) (2250 vs. 6658; Mann-Whitney: *P *= 0.034). Only four patients with a tumor size of more than five centimeters (>5 cm) were recruited. There was no significant difference in the level of ccf nDNA between patients with a tumor size of more than five centimeters (>5 cm) and a tumor size from 2 to 5 centimeters (2 cm-5 cm). No correlation between the levels of ccf mtDNA and tumor size could be found. The correlation between ccf nDNA level and the tumor size is depicted in Fig. 2.

### Association between plasma ccf DNA level and lymph node involvement, extent of metastasis, receptor status of ER, PR and Her2/neu amplification in the malignant disease group

In the malignant disease group no statistical significance in the level of ccf nDNA nor mtDNA between node negative and node positive patients, extent of metastasis and receptor status of ER, PR and Her2/neu amplification could be found.

### The applicability of plasma ccf nDNA and mtDNA as marker for the discrimination between the three study groups

To evaluate the applicability of ccf plasma nDNA and mtDNA as a marker for distinguishing between malignant disease group, benign disease group and healthy control group, we performed ROC (Receiver Operating Characteristic) curve analysis. For the identification of the optimal cut-off point we used the Youden index (J). J is the maximum vertical distance between the ROC-curve and the diagonal reference line and is defined as J = maximum (sensitivity) + (specificity) - 1. The Youden index allows the selection of an optimal cut-off point under the assumption that sensitivity and specificity are equally weighted [18].

### ROC curve analysis using ccf nDNA for the discrimination between the malignant disease group and the healthy control group

**Figure 3:**
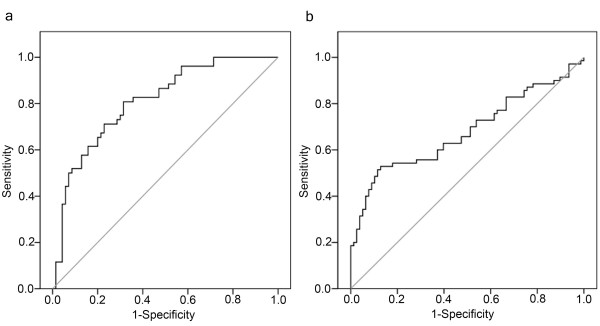
**ROC curves using ccf nDNA and mtDNA for discriminating between the study-groups**. A) ROC curve of ccf nDNA for discriminating between the cancer group and the healthy control group (sensitivity = 81%; specificity = 69%). B) ROC curve of ccf mtDNA for distinguishing between the tumor group and the healthy control group (sensitivity = 53%; specificity = 87%).

Level of ccf nDNA in the malignant disease group was significantly higher in comparison with the healthy control group, but no significant difference was found in the level of ccf nDNA between the benign disease group and the healthy controls. For discriminating between the malignant disease group and the healthy control group, an optimal cut-off point was indicated at 1866 GE/ml for plasma ccf nDNA with a sensitivity of 81% and a specificity of 69% (AUC = 0.80, *P *< 0.001, 95% confidence interval = 0.732-0.885). The ROC-curve for discrimination between the malignant disease group and the healthy control group using ccf nDNA is shown in Fig. 3.

### ROC curve analysis using ccf mtDNA for the discrimination between the breast tumor group and the healthy control group

Decreased levels of ccf mtDNA was found in both the benign disease group and the malignant disease group when compared to the healthy control group. For discriminating between the breast tumor group (malignant and benign) and the healthy control group an optimal cut-off point was indicated at 463282 GE/ml for ccf nDNA with a sensitivity 53% and a specificity of 87% (AUC = 0.68, P < 0.001, 95% confidence interval = 0.589-0.768). The ROC-curve for discrimination between the breast tumor group and the healthy control group using ccf mtDNA is shown in Fig. 3.

## Discussion

According to our knowledge, our study is the first to find increased levels of ccf nDNA and simultaneously decreased levels of ccf mtDNA in plasma samples from patients with breast tumor compared to healthy controls. The former shows a probable diagnostic value in discriminating between breast cancer and healthy controls with a sensitivity of 81% and specificity of 69%, the latter reveals possible relevance in distinguishing between breast tumors (malignant and benign) and normal controls with a sensitivity of 53% and specificity of 87%.

For ccf nDNA, our previous studies indicated that in comparison with other potential circulating biomarkers involved in malignancy, such as nucleosomes, vascular endothelial growth factor (VEGF) and its soluble receptor (sVEGFR1), the ccf DNA showed more sensitivity and specificity in discriminating between breast cancer and normal controls [19,20]. Recently, Diehl et al, explored the possibility of using ccf tumor derived DNA for the management of colorectal cancer [21]. Patients with detectable ccf tumor DNA suffered from relapse, whereas subjects without ccf tumor DNA did not experience tumor recurrence. The ccf tumor DNA detection seems to be more reliable for predicting relapse than the standard biomarker, carcinoembryonic antigen (CEA), used for the management of colorectal cancer [22]. It was also reported that the levels of ccf DNA could be changed after therapy in breast cancer [23,24]. The observations suggest that determination of ccf DNA in cancer may prove a useful tool in the management of the condition.

In this study, we found high levels of ccf plasma DNA related to tumor size. This finding can be supported by investigations in the field of prenatal medicine. Placenta has been regarded as "pseudomalignant" and placental derived ccf fetal DNA in maternal circulation can be used for risk-free prenatal diagnosis [25-27]. The concentration of placental derived ccf fetal DNA in maternal blood increases with the progress in gestational weeks and with respect to placental size [28]. Using fetal specific DNA sequences, ccf fetal DNA could be detected from the 5th gestational week, and the results were reliable by the 8th gestational week with an accuracy of 100% in fetal DNA determination [29,30]. The results imply that by using tumor specific genetic alterations as marker, tumor derived ccf DNA may be detectable at an early stage with confined tumor growth and size.

For mtDNA, both down-or up-regulation in cancer patients has been shown in the past, and many attempts to explain both events have been made. While up-regulation of mtDNA in cancer patients was only demonstrated in a few cases [31], many studies including this one found decreased mtDNA levels in cancer patients [32,33]. One explanation for lower mtDNA copy numbers in cancer patients might be ascribed to mutations or deletions occurring as a consequence of exposure of mtDNA to reactive oxygen species (ROS) which are a by-product of respiration and oxidative phosphorylation. Especially in the D-Loop region which controls replication and transcription of mtDNA, such mutations and deletions may lead to changes in transcription and replication rate and finally result in a decrease of mtDNA levels in cancer patients [34]. In this study we found lower levels of mtDNA in the benign group when compared with the cancer group. In benign tumors depletion of mtDNA could be a mechanism of tumor cells to escape apoptosis and to finally promote cancer progression [35]. On the other hand, the relative increase of mtDNA levels in the cancer group compared to the benign disease group might be a compensatory mechanism of the cells to respond to the decline in respiratory function [36].

We showed that levels of ccf nDNA where significantly elevated in breast cancer patients in comparison with a benign disease group and a healthy control group, while levels of ccf mtDNA were significantly elevated in the breast tumor group (malignant and benign) when compared to the healthy control group. Regarding ccf nDNA levels, our results are confirmed by the findings of other studies which also found altered levels of ccf nDNA in cancer patients. For ccf mtDNA however, both down- as well as upregulation of ccf mtDNA levels in cancer patients have been reported and therefore grant further investigations of mtDNA content in different cancer and tumor types, in order to clearly establish whether mtDNA levels are cancer type or tumor specific.

To conclude, both ccf nDNA and mtDNA levels allowed for discrimination between the different study groups. While ccf nDNA could be used for discriminating between patients with breast cancer and healthy controls, ccf mtDNA could be used for distinguishing between patients with breast tumors (malignant and benign) and healthy controls. Altogether this suggests that ccf nDNA has potential as a cancer specific biomarker, whereas ccf mtDNA may rather serve as a tumor biomarker.

## Competing interests

The authors declare that they have no competing interests.

## Authors' contributions

CK carried out the simultaneous quantification of ccf nDNA and mtDNA from plasma as well as the statistical data analysis and drafted the manuscript. RR, ZB and RA participated in data analysis and helped to draft the manuscript. NB and CD were responsible for the patient recruitment and the clinical study. JB, EW, WH and XYZ participated in the design and in the coordination of the study. All authors read and approved the final manuscript.
